# Application of the world guidelines for falls prevention and management’s risk stratification algorithm to patients on a frailty intervention pathway and the potential utility of sensory impairment information

**DOI:** 10.1186/s12877-024-05405-3

**Published:** 2024-10-12

**Authors:** Roulla Katiri, Jack A. Holman, Siobhán Magner, Cian O’Caheny, Colm P. Byrne

**Affiliations:** 1 Audiology Department, Mater Misericordiae University Hospital, Dublin, Ireland; 2 Hearing Sciences, Mental Health and Clinical Neurosciences, School of Medicine, University of Nottingham, Nottingham, UK; 3 Adult Diagnostic Audiology, University College London Hospitals NHS Foundation Trust, Royal National ENT & Eastman Dental Hospitals, London, UK; 4 Hearing Sciences (Scottish Section), Mental Health and Clinical Neurosciences, School of Medicine, University of Nottingham, Glasgow, UK; 5 Physiotherapy Department, Mater Misericordiae University Hospital, Dublin, Ireland; 6 Pharmacy and Medicines Optimisation Directorate, Mater Misericordiae University Hospital, Dublin, Ireland; 7 Department of Geriatric Medicine, Mater Misericordiae University Hospital, Dublin, Ireland

**Keywords:** Elderly, Falls, Sensory function, Frailty, Falls prevention

## Abstract

**Background:**

The 2022 world guidelines for falls prevention and management suggest measuring sensory function including dizziness, vision, and hearing. These variables are not included in the falls risk stratification algorithm. This study sought to investigate the utility of the guidelines and potential avenues for improvement. This study applied the falls risk stratification recommendations and reviewed the individual sensory impairment risk factor variables predictive of falls and falls risk grouping in those assessed by a frailty intervention team (FIT) based in an emergency department (ED).

**Methods:**

Patients over 65 years old who attended the ED and had a comprehensive geriatric assessment carried out by FIT over a period of four months were included in this retrospective cross-sectional study. Patient characteristics, medication, physical and sensory function status data was retrieved and analysed with respect to falls and falls risk grouping.

**Results:**

Data was gathered retrospectively from 392 patients. Excluding those with missing data, almost all attendees were in the high-risk of falls category (*n* = 170, 43.4%), or the low-risk category (*n* = 149, 38.0%). Few people were in the intermediate-risk category (*n* = 19, 4.8%). Hearing loss and dizziness were significantly associated with falls incidence, whereas vision and balance were not. Hearing loss, balance and dizziness were significantly associated with risk grouping, whereas vision was not.

**Conclusions:**

Most older adults included in the analysis fell into the low- or high-risk categories, with a minority in the intermediate-risk category. This suggests that the inclusion criteria for the intermediate category could be altered for greater sensitivity. While impaired balance and vision were the most common impairments, hearing status, balance and dizziness were associated with risk group. These results, through a practical application of the world guidelines for falls to an acute clinical sample, raise the possibility of refining the falls risk stratification criteria, and highlight the capacity for additional sensory intervention to mitigate falls risk.

**Supplementary Information:**

The online version contains supplementary material available at 10.1186/s12877-024-05405-3.

## Background

A fall is defined as ‘*an event which results in a person coming to rest inadvertently on the ground or floor or other lower level. Falls*,* trips and slips can occur on one level or from a height*’ [1]. Falls are the second largest cause of unintentional injury related deaths worldwide [2]. It is estimated that approximately 684,000 people die from falls annually, of which the majority occur in low- or middle-income countries [3]. The World Health Organisation (WHO) estimate that the cost of falls is in the region of €400 billion annually [4].

In the United Kingdom it is estimated that 30% of people aged 65 years or older, will fall at least once a year; and this increases to 50% for those aged over 80 years [5]. Two thirds of this group are likely to have a subsequent fall in the following 12 months [6]. The NHS in England estimate that the cost of falls is in the region of £435 million [5]. Similarly, in Ireland annual hospital admissions due to falls have been estimated in 2006 to be €10.8 million [7].

The recently published world guidelines for falls prevention and management [1] provide a patient-centred framework to support the identification, prevention, and management of falls in older adults. The guidelines describe a stratification algorithm for low-, intermediate-, or high-risk of falling. The rationale is for healthcare professionals to use the stratification tool to proactively identify the falls risk in older adults, and therefore prevent and manage the likelihood of occurrence of falls. The guidelines suggest measuring sensory function with regards to falls risk factors including dizziness, vision, and hearing, although these are not included in the risk stratification algorithm.

The Irish Longitudinal Study on Ageing (TILDA) have recently implemented the framework in an observational study aiming to successfully differentiate those at greater risk of falling [8]. The TILDA analysis included a non-clinical group of older adults and most of their participants were classified in the low-risk category. They found very little difference between intermediate- and high-risk groups, with less than 1% of their participants falling into the intermediate group. The reasons for the majority of patients falling into the low- or high-risk categories was attributed to the non-clinical nature of the TILDA participant sample. Therefore, the authors suggested further feasibility assessment of the of the algorithm with other population samples, such as a group of patients from a clinical setting.

A Frailty Intervention Team (FIT) was established in October 2020, in the emergency department, at a large level 4 teaching hospital in Dublin’s north inner city. The FIT is an interdisciplinary group consisting of physiotherapy, occupational therapy, speech and language therapy, dietetics, medical social work, pharmacy, nursing, therapy assistants, and medical team members. They complete comprehensive geriatric assessments (CGAs) for older adults, aged 65 years or older, that attend the emergency department. CGAs are completed by all members of the FIT at any stage and/or throughout their attendance at the emergency department.

CGAs are utilised as a multidisciplinary diagnostic process to help the FIT identify the medical, psychosocial, and functional needs of older adults. There is strong evidence that in the medical setting, CGAs can guide the development of a coordinated plan to manage the health complexity, and to maximise overall health in older persons [9]. The development of a falls risk stratification algorithm is a potentially valuable tool to complement, or to be integrated into, existing treatment protocols or assessments such as the CGA. While the information provided in the world guidelines for falls prevention and management [7] is comprehensive, there is also a question as to whether other information, such as that gathered by CGAs, could enhance the utility of falls guidelines or risk stratification algorithms.

There is evidence highlighting that sensory issues are linked to an increased risk of falls. Particularly in older adults, hearing loss [10, 11], vision loss [12], and balance [13], have been identified as related to falls. The presence of dizziness can also be associated to falls, particularly in older females [14], or fear of falling [15]. Alterations or deterioration in sensory function, such as visual or auditory impairment, are associated with increased risk of falls, and dual sensory impairment may exacerbate the risk of falling due to inability to compensate with sensory substitution [16]. Although the underlying mechanisms for such relationships are still to be fully elucidated [17], hypothetically, interventions to treat sensory issues should reduce falls risk, so assessment of sensory issues as part of falls risk assessment could be of great importance.

The primary aim of this study was to assess the utility of applying the world guidelines for falls prevention and management stratification algorithm [1] to older adults presenting to the emergency department, who have had a CGA completed. The secondary aim was to identify any relationship between sensory variables such as those gathered by the CGA and falls risk stratification of patients who were seen on a FIT pathway.

## Methods

The study was a retrospective cross-sectional assessment of patient data over a defined period of time. The primary research questions were:

(i) Can the world guidelines for falls prevention and management stratification algorithm be successfully utilised in an emergency department setting?
(ii) Are sensory variables related to falls and falls risk stratification?
(iii) What other variables gathered by a CGA could inform the assessment of, and interventions for, falls risk?

**Fig. 1:**
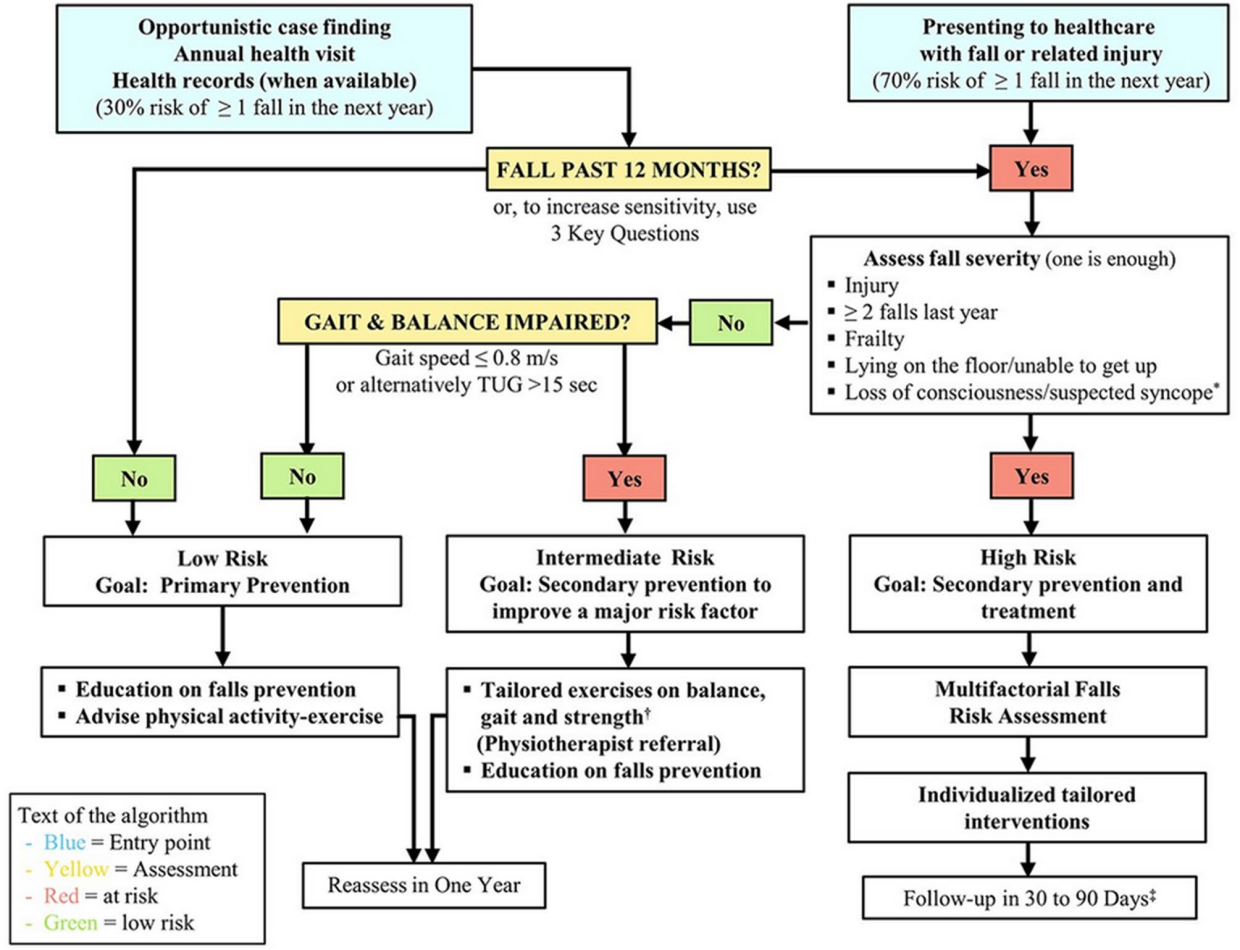
World guideline for falls prevention and management algorithm for risk stratification, assessments and management / interventions for community-dwelling older adults [1]. CC-BY-NC

The authors used a form of convenience sampling. It was decided that a minimum of 200 patients would be needed to give a meaningful insight into the utility of falls guidelines stratification in this observational study. Following an assessment of a random two-week time window of the number of patients who had a CGA carried out, it was established that three months’ worth of data would be adequate accounting for patterns of missing data. Therefore, all patients aged 65 years or older, who attended the emergency department at the Mater Misericordiae University Hospital and who had a CGA carried out by the FIT between January 3rd and April 20th 2023 were included in the study. Patients younger than 65 years old, and those who attended the emergency department in the specified study period, but did not have a CGA completed by the FIT were excluded. Records were gathered by three of the authors (COC, SM, RK), who are clinicians authorised by the clinical audit and effectiveness committee to access and handle data. In cases where the patient was unable to provide information about their health (e.g., in cases of cognitive impairment), information was gathered from family members and/or carers, as appropriate, or from existing medical records (e.g., previous hearing test results saved on the patient management system). All participant records were pseudonymised before analysis. Sensory function status data was retrieved from a detailed CGA form completed for patients seen. Fall-risk-increasing drugs (FRIDs), as defined by Screening Tool of Older Persons Prescriptions in older adults with high fall risk (STOPPFall) [18], was retrieved from a pre-admission medicines reconciliation performed by a pharmacist. The majority of participants were community-dwelling adults, although the CGA did not specifically collect information on the participants’ domestic status. The study applied the falls risk stratification recommendations from the world guidelines for falls prevention and management [1, Fig. 1] using information gathered from the CGA form (additional file 1), and reviewed the individual risk factor variables predictive of falls in those accessed.

The study protocol was reviewed and approved by the clinical audit and effectiveness committee, Quality and Patient Safety Directorate, at the Mater Misericordiae University Hospital, Dublin (Reference number: CA23-011, approved 22/02/2023).

The world guidelines for falls prevention and management [1] suggest risk groupings based on multiple factors (Fig. 1). The initial factor is having had one or more falls in the past 12 months, for which the CGA gathers data. Those who are a ‘no’ for this are listed as low risk. All others are assessed further. Patients are classified as high risk if they subsequently meet at least one of five falls severity criteria: (i) greater than or equal to two falls in the past year, (ii) frailty, (iii) lying on the floor unable to get up, (iv) loss of consciousness, or (v) injury. The CGA gathers data on the first two factors (number of falls and frailty), but not on the other three factors. Patients who do not meet any of the five mentioned criteria are then assessed for grouping in the low- or intermediate-risk categories. Intermediate risk is assigned if patients have either impaired balance or impaired gait, otherwise low risk is assigned. The CGA gathers data on balance but not gait speed. Consequently, the data from the CGA can be used to place patients into risk groupings in an informed, but not perfect, manner.

The raw data was coded for analysis in the following way: Gender (female = 1, male = 2), History of falls in last 12 months (no = 0, yes = 1), Clinical frailty score [19; additional file 2] (1 very fit-9 terminally ill; 1–4 = low frailty, 5–9 = high frailty), vision (intact = 0, impaired = 1), hearing (intact = 0, impaired = 1), dizziness (no = 0, yes = 1), balance issues (no = 0, yes = 1), glasses (no = 0, yes = 1), hearing aids (no = 0, yes = 1), and mobility aid (unaided = 0, aided = 1). Missing data was coded N/A and patients with missing data for any given key criteria were excluded from the corresponding analysis. Binomial logistic regression was used to assess the relationship between sensory variables (vision, hearing, dizziness, and balance), falls and falls risk stratification. The regression controlled for the effect of age, sex, and frailty. The statistical software R [20] was used to analyse the data. The Strengthening the Reporting of Observational Studies in Epidemiology (STROBE) checklist has been utilised for reporting the study findings (additional file 3) [21].

## Results

In total, data was gathered retrospectively from 392 patients, 227 (57.8%) female, and 166 male. The mean age was 82 years old, with a range of 65 to 103 years. In total 134 (34.2%) patients presented at the emergency department with a fall, 255 (65.1%) presented for another reason and three records had missing data for presentation. Over half (*n* = 223, 56.9%) reported a fall in the past year, 138 (35.2%) had no recent falls, and 31 (8.0%) had missing data on falls. The mean Clinical frailty score was 5 (mildly frail). Table 1 presents the demographic data of all patients as well as those for whom falls risk classification was possible (i.e., where there was not missing data that made classification impossible).

**Table 1:** Patient demographics

	All patients (*n* = 392)	Classifiable for falls risk (*n* = 338)
Number female (%)	227 (58%)	200 (59%)
Age range (years)	65–103	65–103
Age mean (years)	82.2	82.5
Mean clinical frailty score (excluding missing data)	5.13	5.18
Reported a fall in the past year (excluding missing data)	198	183
Presented with a fall (excluding missing data)	134	120

### (i) Can the world guidelines for falls prevention and management risk stratification algorithm be successfully utilised in an emergency department setting?

#### Falls risk stratification

**Fig. 2:**
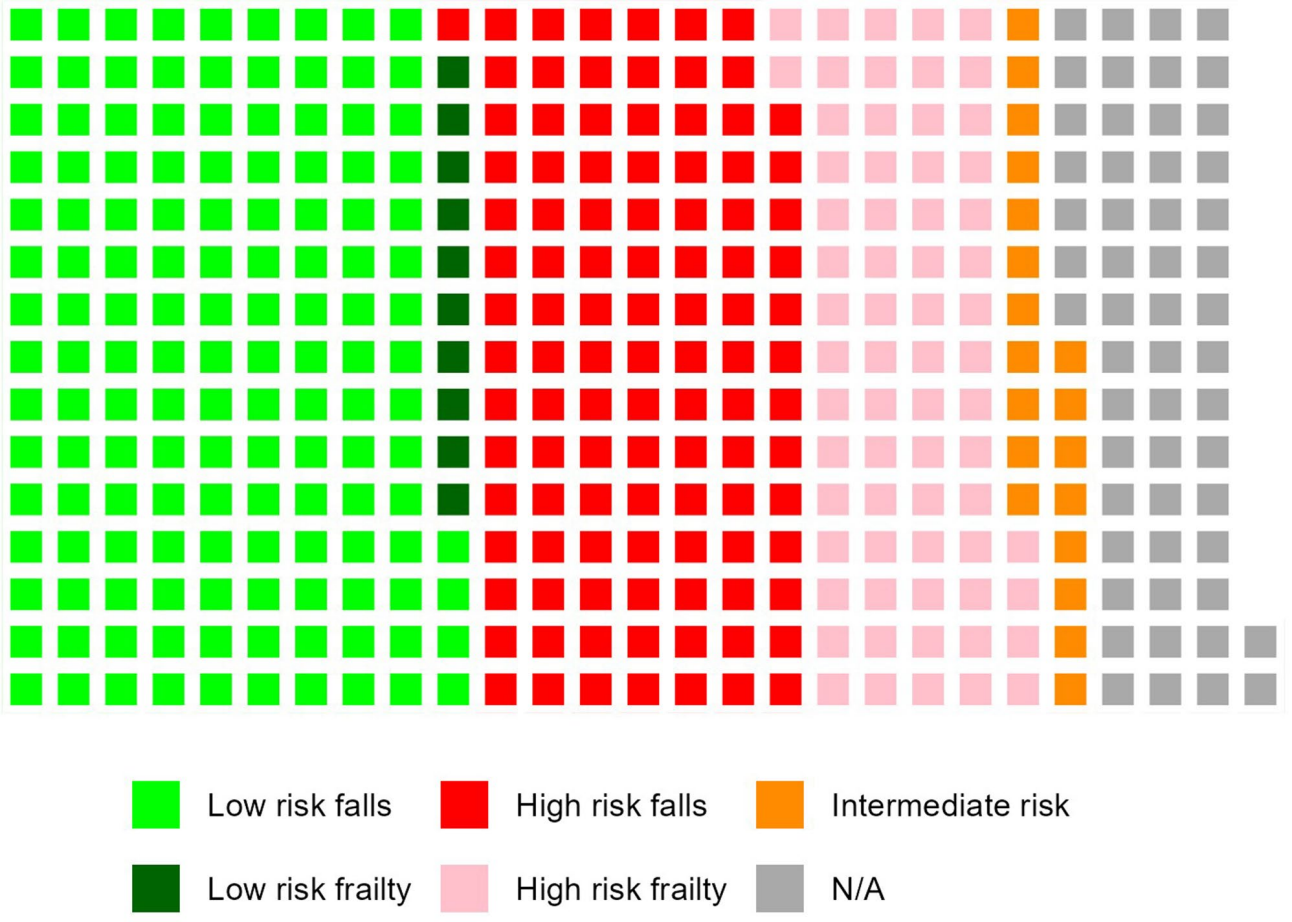
Waffle plot of risk stratification based on world guidelines for falls prevention and management. Low risk falls = less than one fall in past year; Low risk frailty = one fall in past year but no frailty or balance issues; High risk falls = greater than or equal to two falls in the past year; High risk frailty = one fall in the past year and frailty; Intermediate risk = one fall in the past year, balance issue and no frailty; N/A = not able to stratify patient because of missing data

Of those who presented, the majority (*n* = 170, 43.4%) fitted the high-risk category criteria (Fig. 2). One hundred and four (26.6%) were classified as high risk due to greater than or equal to two falls in the past year. Sixty-six (16.8%) were classified as high risk due to having had one fall in the past year and a high frailty score. Approximately one third of patients (*n* = 149, 38.0%) fell into the low-risk category. Of those, 139 (35.4%) patients had no falls in the past year, while 10 (2.5%) had one fall in the past year, low frailty scores, and no balance issues. Only a very small minority (*n* = 19, 4.8%) of those who were assessed were categorised as having an intermediate risk. Due to incomplete raw data some patients (*n* = 54, 13.8%) could not be categorised.

### (ii) Are sensory variables related to falls and falls risk stratification?

#### Relationship between sensory variables and falls

The relationships between the sensory variables vision loss, hearing loss, dizziness and balance issues and falls incidence in the past year were individually calculated, controlling for the influence of age, sex, and frailty. Hearing loss was a significant predictor of falls incidence, b = 0.68, z = 2.4, *p* = 0.016. Presence of hearing loss was significantly associated with a 1.97 greater likelihood of falls incidence in the past year. Dizziness was a significant predictor of falls incidence, b = 0.81, z = 2.18, *p* = 0.027. Presence of dizziness was significantly associated with a 2.24 greater likelihood of falls incidence in the past year. There was no significant association between vision loss (b = 0.33, z = 1.26, p = > 0.05) or balance issues (b = 0.34, z = 0.96, p = > 0.05) and falls incidence. Neither model was improved by introducing the interaction effect of sensory variable and age. While the significant relationships between the sensory variables of hearing loss and dizziness and falls are informative and valuable, as expected each model explains only a small amount of the total variance in falls as measured by pseudo-r-squared values (Nagelkerke R^2^ values of 4.5% and 3.7% respectively).

#### Relationship between sensory variables and falls risk stratification

**Fig. 3:**
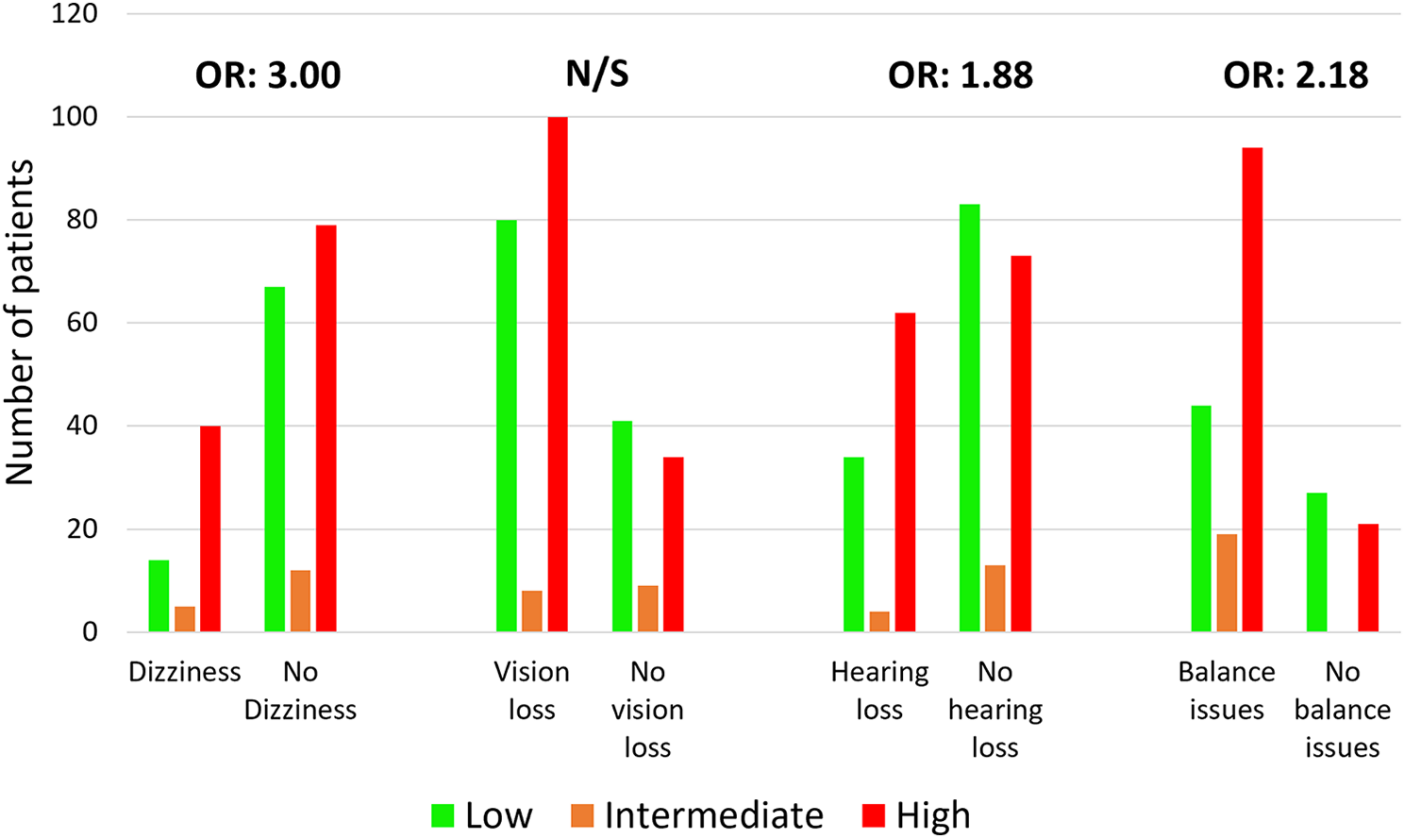
Existence of sensory issues in each of the three risk groups following stratification. Odds ratios (OR) are the odds of being in the high-risk group compared to the low-risk group based on having versus not having the sensory issue. The odds ratio for each sensory issue in turn accounts for the effect of age, sex and frailty but not the effect of other sensory issues

In the low-risk group, the most common sensory issue was vision loss (*n* = 80, 66.1%) followed by balance issues (*n* = 44, 62.0%). In the high-risk group, the most common sensory issue was also vision loss (*n* = 100, 74.6%) followed by balance issues (*n* = 94, 81.7%). Percentages were calculated after removing those for whom there was missing data for that sensory variable. Hearing loss was a significant predictor of falls risk grouping, b = 0.63, z= -0.76, *p* = 0.028. Hearing loss was significantly associated with a 1.88 greater likelihood of being classified as high risk for falls (Fig. 3). Dizziness was a significant predictor of falls risk grouping, b = 1.1, z = 2.95, *p* = 0.003. Dizziness was significantly associated with a 3.0 greater likelihood of being classified as high risk for falls. Balance issues were a significant predictor of falls risk grouping, b = 0.78, z = 2.16, *p* = 0.03. Balance issues were significantly associated with a 2.18 greater likelihood of being classified as high risk for falls. Vision loss was not a significant predictor of falls risk grouping, b = 0.47, z = 1.63, p = > 0.05.

### (iii) What other variables gathered by a CGA could inform the assessment of, and interventions for, falls risk?

#### Interventions for sensory issues

**Fig. 4:**
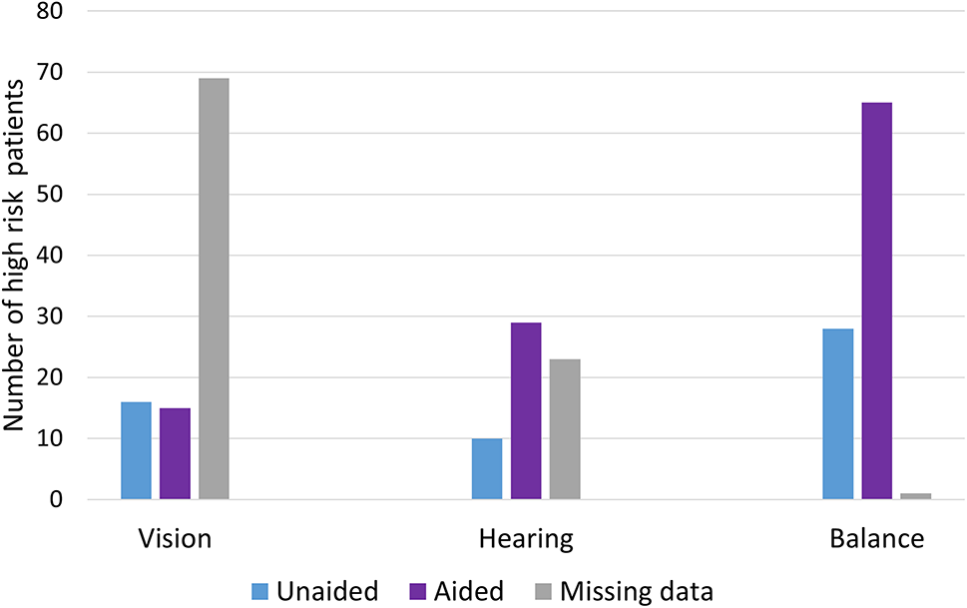
Number of high-risk patients experiencing vision loss, hearing loss and balance issues who are aided and not aided for the condition

The CGA gathered information on interventions for hearing loss (hearing aids), vision loss (glasses) and balance issues (mobility aids). The results show that a majority of patients in the high-risk falls group with these sensory issues have already been given interventions. Discounting those for whom there is missing intervention data, in the high-risk falls group 74% of those with hearing loss had a hearing aid, 48% of those with vision loss had glasses and 70% of those with balance issues had mobility aids. There was a large amount of missing data for vision loss in particular (69%). There was missing intervention data of 37% and 1% for hearing and balance respectively. The figures mean there is some limited scope for dealing with untreated sensory problems. (Fig. 4).

#### Falls-risk-increasing drugs and other variables gathered by the CGA

Pre-admission medicines reconciliations data was available for 94 participtants in our cohort. In the low-risk group (*n* = 23) 17 patients (73.9%) took falls risk inducing medication (FRID). In the intermediate-risk group (*n* = 8) two patients (25%) took FRID. In the high-risk group (*n* = 63) 52 patients (82.5%) took FRID. Subsequently five patients were de-prescribed FRID in the low-risk group and nine were de-prescribed FRID in the high-risk group. Of our sample, four patients were on five or more FRIDs. FRID use was a significant predictor of falls risk grouping, b = 1.15, z = 3.74, p = < 0.001. FRID use was significantly associated with a 3.15 greater likelihood of being classified as high risk for falls. Other variables gathered as part of the FIT assessment such as cardiovascular risk and delirium were not related to falls or falls risk grouping.

## Discussion

The study results showed that the world guidelines for falls prevention and management’s risk stratification algorithm can be successfully utilised in a clinical population, sensory variables hearing loss and dizziness were significantly associated with falls incidence and hearing loss, balance and dizziness were significantly associated with risk grouping. In keeping with the findings of Hartley et al. (2023), who applied the stratification to a non-clinical population [8], the majority of patients fell into the low- or high-risk groups with less than 1% falling into the intermediate group. Only 4.8% of our sample were classified as intermediate risk, which raises the question of why such a low proportion was identified. It is possible that the non-clinical population of Hartley and colleagues and the acute clinical population in the present study are not where those of intermediate risk would be identified. In this study we used impaired balance as the metric for intermediate group classification in lieu of TUG scores and gait speed. It is possible that the inclusion of these scores could have increased the number of patients in the intermediate risk group, however this would have only been by a maximum of ten patients which would not constitute a meaningful change to the conclusions. Alternatively, it is possible that changes to the stratification algorithm could result in greater sensitivity and therefore more utility. For example, such changes could involve the addition of relevant sensory variables or altering the inclusion and/or exclusion criteria for risk groups.

The study population consisted of older adults who were taken through a frailty screening. Frailty has been connected to the sensory variables of hearing loss [22], vision loss [23] and balance [24], and the variable of falling itself [25]. Furthermore, older adults with co-existing hearing and visual impairment could be more prone to falls [26]. This is mainly due to the inability of people with dual impairment to utilise compensatory information about their body posture or their environment from other sensory inputs [16]. As such, this research sought to control for the effect of frailty when analysing the impact of each sensory variable on falls risk. Many links have been made between different sensory variables and falls risk. A meta-analysis by Jiam and colleagues (2016) found that older adults with hearing loss were between 1.72 and 2.39 times more likely to fall than those without hearing loss [27]. Our finding of 1.97 greater likelihood of falls is in keeping with such findings. There is further evidence to suggest that hearing loss leads to poorer pitch perception [27], and that increased cognitive load caused by hearing loss can result in poorer postural control [28], both of which increase the likelihood of falls. Vision loss has been linked to falls, particularly due to environmental hazards which might otherwise have been avoided [12]. Understandably, there is also a wide literature relating both balance [13] and dizziness [28] to falls risk. One study limitation is that the CGA did not collect information about the participants’ living situation (e.g., community dwelling, or living at a residential care facility). Residents of care facilities are expected to be frailer, and a metanalysis has recently demonstrated that frailty in those aged 65 years or older is associated to a higher risk of falls [29].

Sensory interventions, such as hearing aids, have been shown to be related to a reduction in falls amongst older adults [30]. In our sample we assessed the scope for providing sensory interventions as a means of reducing falls risk. Unfortunately, there was a large amount of missing data when it came to interventions for vision loss (such as glasses), and a smaller amount of missing data for hearing interventions. Regardless, our sample included individuals experiencing each sensory issue who had not yet received an intervention, suggesting that there is scope for further intervention, even if not for a majority of individuals.

This study analysed data from 392 patients, however many patients’ CGAs were missing data for one or more variables. This meant that analysis controlling for the effects of other target variables was not possible given the increasingly small numbers of patients with complete data. Future research should analyse larger numbers of patients to assess the comparative impact of the target sensory variable given the presence or non-presence of other sensory impairments. This is also the case for other influential variables such as falls risk inducing medication and polypharmacy. Polypharmacy in particular is linked to a 21% increase in the rate of falls over a two-year period [31], and in this study we identified four patients who were taking five FRIDs even before inclusion of other non-falls risk inducing medication. However, resource limitations during the period examined meant pre-admission medication data via medicines reconciliation was only available for 94 participants. As mentioned previously, it is possible that changes to the stratification algorithm could improve its effectiveness in accurately assigning nuanced and meaningful risk classifications. For example, the results of this research suggest that hearing loss and dizziness are independently related to falls in the past year. There were 47 individuals in the low-risk group with either a hearing loss or dizziness and there may be grounds for including such individuals in the intermediate-risk group. Future research should further investigate the various sensory variables know to be related to falls and exactly how their inclusion would influence the stratification algorithm. The development of the world guidelines for falls prevention and management is an important step forward for falls care, and clinical practice will likely change to incorporate the guideline’s suggestions. The results from this study are important as they identify areas where further development can be made for increased patient benefit.

## Conclusion

The world guidelines for falls prevention and management’s risk stratification algorithm can be utilised clinically, even in a sample of patients on a frailty intervention pathway which was designed before publication of the guidelines. Using the risk stratification algorithm, most of the included patients fell into either the high- or low-risk groups as has been shown in non-clinical populations. Only 4.8% of those stratified were categorised as intermediate risk. This may be accounted for due to the relatively frail nature of the cohort included but is similar to results from a non-clinical sample. Those with hearing impairment, balance issues, and dizziness, independent of frailty, age, and gender, were more likely to be categorised as high risk. This demonstrates the influence of sensory loss in falls risk, highlighting the potential importance of sensory assessment as part of a multi-factorial fall prevention strategy in older adults. Such improvements which utilise sensory impairment information could lead to earlier intervention and better health outcomes.

## Electronic supplementary material

Below is the link to the electronic supplementary material.

Supplementary Material 1

Supplementary Material 2

Supplementary Material 3

## Data Availability

The raw data are not publicly available but can be made available upon request to the corresponding author.
