# Supplementary material for: Application of the world guidelines for falls prevention and management’s risk stratification algorithm to patients on a frailty intervention pathway and the potential utility of sensory impairment information
Source: BMC Geriatr. 2024 Oct 12;24:824. doi: 10.1186/s12877-024-05405-3 (PMC11470725; doi:10.1186/s12877-024-05405-3)
Supplement: Supplementary file 1 — Supplementary Material 1 [file 12877_2024_5405_MOESM1_ESM.doc]

| **Client**  **Name:**  **MRN:**  **Address:**    **DOB:**  **Known as:** | | | | | | | **Alerts:**  **Delirium/Dementia**  **Incontinence**  **Polypharmacy**  **Assistance with Personal Care**  **Injuries/Falls risk Hx**  **Assistance with Mobility**  **Malnourished/Supplements**  **Modified Diet**  **Clinical Frailty Scale: (specify)**  **Pressure Risk or Injury: (specify)**  **Safeguarding** | | | | | | |
| --- | --- | --- | --- | --- | --- | --- | --- | --- | --- | --- | --- | --- | --- |
| Consent obtained to AX. And sharing details **Y**  **N** | | | | | | | ED/AMAU attendance in the past 3 months **Y**  **N** | | | | | | |
| **Assessor Designation** | |  | | | | | Hospitalisation (in-patient) in the last month **Y**  **N** | | | | | | |
| **Date:** | | **Time Screen started:** | | | | | **Supports and Contacts** | | | | | | |
| **Presenting Complaint** | | | | | | | **Patient Contact No:** No Landline, | | | | | | |
|  | | | | | | | **Main Contact**: | | | | | **Relationship:** | |
|  |  |  |  |  |  |  | **Phone:** No Landline | | | | | | |
|  |  |  |  |  |  |  | **Medical Card Number:** | | | | | | |
|  |  |  |  |  |  |  | **Formal Supports:** HH HCP Private Care | | | | | | |
|  |  |  |  |  |  |  | **Details (name of Agency, no. of visits, etc.)** | | | | | | |
|  |  |  |  |  |  |  | **Informal Support** | | | |  | | |
| **Past Medical/ Surgical History** | | | | | | | **MOW** | | | |  | | |
|  | | | | | | | **Day Centre** | | | |  | | |
|  |  |  |  |  |  |  | **CMHT** | | | |  | | |
|  |  |  |  |  |  |  | **PHN** | | | |  | | |
|  |  |  |  |  |  |  | **Other PCC:** OT  Physio  SLT  Dietician MSW | | | | | | |
|  |  |  |  |  |  |  | **Language** | | | |  | | |
| **Medical Investigations and Observations** | | | | | | | | | | | | | |
| **Bloods**: | | | | | | | | | | | | | |
| **Imaging**: | | | | | | | | | | | | | |
| **Other**: | | | | | | | | | | | | | |
| **IPC Status:** | | | | | | | | | | | | | |
| **Pain:** | | | | Location, aggravating factors, analgesia | | | | | | | | | |
| **Pressure Injuries:** | | | | **Y**  **N** Details: | | | | | | Current Pressure Care: Cushion  Mattress Details: | | | |
| **Psychosocial/Mood:** | | | | Do you feel depressed or low? **Y**  **N** | | | | | | Are you lonely/isolated? **Y**  **N** | | | |
| **Vision:** | | | | Intact  Impaired Details: | | | | | | Test in last year **Y**  **N** Bifocals **Y**  **N** | | | |
| **Hearing:** | | | | Intact  Impaired Details: | | | | | | Hearing Aids **Y**  **N** Wearing **Y**  **N** | | | |
| **Sleeping Pattern** | | | |  | | | | | | | | | |
| **Recent behaviour change/Agitation/Aggression** | | | | (specify) | | | | | | | | | |
| **Self-Neglect/Wandering** | | | | (specify) | | | | | | | | | |
| **Safety Issues or Concerns** | | | | (specify) | | | | | | | | | |
| **Living Arrangements** | | | | | | **Housing** | | | | | | | |
| Lives alone with Spouse  Other  ______________  Private Ownership  Private Rental  Council Tenant  Sheltered Housing  Name: ________________  Nursing Home  Name: ___________________ | | | | | |  | | Bungalow  2 Storey  3 Storey  Apartment  Floor No: ____ Lift **Y**  **N**  Smoke Alarm **Y**  **N**  Carbon Monoxide **Y**  **N**  Mobile phone **Y**  **N** Carries ___________  Pendant Alarm  Wearing Pendant  None | | | | | |
| **Stairs** | | | | | | **Personal Care** | | | | | | | |
| 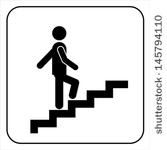 | One Banister  Left  Right  Ascending Two banisters/2^nd^ handrail  Number of steps to front door: _______  Number of steps to back door: _______  Internal steps: **Y**  **N**  **_________**  Stairlift : **Y**  **N** | | | | | 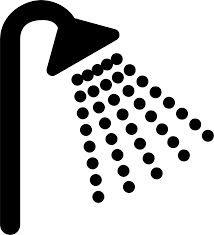 | | Bedroom: Upstairs  Downstairs  Toilet: Upstairs  Downstairs  Bath: Upstairs  Downstairs  Shower: Upstairs  Downstairs  Seat  Level Access Shower  Over Bath  Step in  Other: (specify) | | | | | |
| **Lifestyle** | | | | | | | | | | | | | |
| 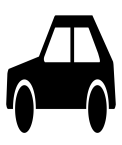 | Means of Transport: (specify)  Does the patient drive: **Y**  **N**  If yes: Locally **Y**  **N**  Long Distance **Y**  **N**  Previous Driving Assessment **Y**  **N** | | | | | Future Planning: EPOA **Y**   **N**  In progress  NHSS Initiated **Y**  **N** | | | | | | | |
|  |  |  |  |  |  | Smoker **Y**  **N**  (no. of cigs/ day): Ex-smoker **Y**  **N**  Alcohol **Y**  **N**  (units per week): | | | | | | | |
| 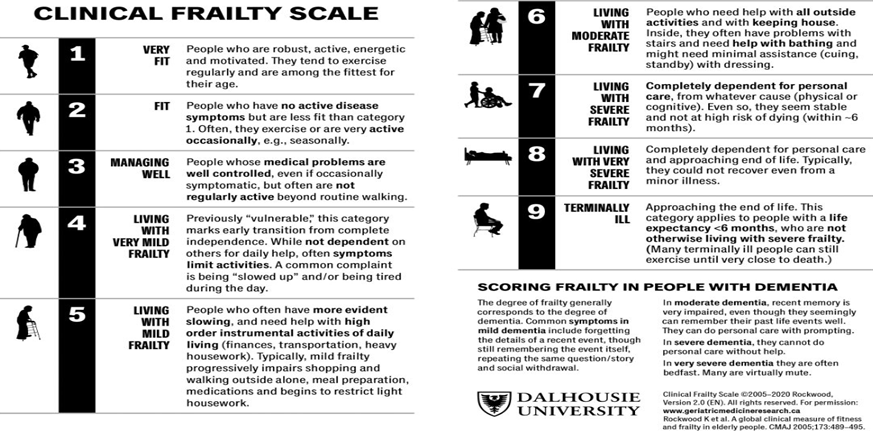 | | | | | | | | | | | | | **CFS Score:** |
|  |  |  |  |  |  |  |  |  |  |  |  |  | **Comment:** |
| **Cognitive Screen** | | | | | | | | | **Pharmacy** | | | | |
| **4AT:** **Assessment for Delirium and Cognitive Impairment**  **1. Alertness:** Normal **0** Mild Sleepiness **0** Abnormal **4**  **2. AMT4:** Age, Date of Birth, Place (Hospital Name), Year  No mistakes **0** 1 Mistake **1** ≥ 2 Mistakes **2**  **3. Attention:** Months of the year backwards  ≥ 7 months or more correctly **0** Starts but scores < 7 months **1** Untestable **2**  **4. Acute Change/fluctuating symptoms** (over the last 2 weeks and still evident in last 24hrs)  NO **0** YES **4**  ***4AT Scoring:* 0** = Normal, **1**=? Mild Cog. Impairment, **2-3**=? Cog. Impairment  **≥ 4 Suspect Delirium +/- Dementia 4AT Score:** | | | | | | | | | Falls **Y**  **N**  Renal Impairment  Epilepsy  Diabetes Parkinson’s  6+ Medications **Y**  **N**  Allergies **Y**  **N**  Blister Pack  Packaging  Difficulty with managing medications **Y**  **N**  Details:  **Medicines Reconciliation Completed** | | | | |
| **Requires further cognitive assessment Y** (refer to OT) **N** | | | | | | | | |  | | | | |
| **Previous Cog Ax: Y**  **N** | | | **Result:** | | **Date:** | | | |  |  |  |  |  |

| **Nutrition, Swallowing and Communication** | | | | | | | | | |
| --- | --- | --- | --- | --- | --- | --- | --- | --- | --- |
| **Nutrition Screen If MST ≥2 or if “Yes” to any of the below refer to the Dietician**  **A. Has the patient lost weight recently?** Yes - Go to **B**  No – Go to **C**  Unsure – Go to **C** + score **2**  **B. Weight Loss score (6 months)** 2-13 lbs (1-5kg) = **1** 1-1.5 stone (6-10kg) = **2** 1.5 -2 stone (11-15kg) = **3** ≥ 2 stone (> 15kg) = **4**   | **Weight:**  **MST Score:** | | --- | | | | | **Speech and Language Therapy Screen**  **Respiratory** COPD  LRTI  Lung Ca  Recurrent Chest Infections  **Neuro** Old CVA  Progressive Neuro (PD, MS, MND, etc.)  Dementia  Chronic use of meds for mental health difficulties  **Does the person report any swallowing difficulties? Y**  **N**  **Fluids:** Level ___ **Diet:** Level ___  **Does the person report any difficulties swallowing tablets? Y**  **N**  **Able to feed themselves independently? Y**  **N**  **Can the person communicate their basic needs (to ask for drinks/food, to ask to go to the bathroom, etc.)?**  **Y**  **N**  **Can you understand their speech? Y**  **N** | | | | | |
| **C. Lack of appetite / eating poorly**  **Yes = 1**  **No = 0**  **MST Score =** Patient admitted with a feeding tube: **Y**  **N**  Patient prescribed Oral Nutritional Supplements: **Y**  **N**  SLT assessment of swallow indicates referral to Dietetics required due to dysphagia: **Y**  **N**  Increased nutritional requirements-pressure ulcer (grades ≥ 2) **Y**  **N** | | | |
| **Falls Screen** | | | | | | **Ax of Orthostatic Hypertension** | | | |
| **1.** In the past year have you had any fall, including a slip or trip in which you lost your balance and lost your balance and landed on the floor or ground or lower level? **Y**  **N** a. How many times did you fall in the past year? _______ b. How did you fall? (please describe e.g. activity, place, time):  C. Could you get up from the floor following your fall? **Y**  **N**  **2.** Are you afraid of falling? **Y**  **N**  Sometimes  Don’t know  **3.** Did your fall require medical intervention? (specify):  **4.** Have you any difficulty with your walking or balance? (specify):  **5.** Do you get Dizzy or light headed? **Y**  **N**  Is this on standing up? **Y**  **N**  **Please complete Ax for Orthostatic Hypotension**  **ECG Done Y**  **N**  **Comments( specify)**:  **Any previous or present bony fracture: Y**  **N**  **If Yes specify**:  **Bone health medication: Y**  **N**  **Type**:  **Footwear**: | | | | | | **Procedure for measuring lying and standing BP.**   - **Select correct cuff size for manual/electronic BP machine.** - **Lie down for 5 minutes. Take BP 1=** - **Stand up. Take BP 2 in 1^st^ minute=** - **After 3 minutes take BP 3=**   **Symptoms (specify)**:  **POSITIVE RESULT Y**  **N**   - **Drop in systolic BP of 20mmHG or more.** - **A drop in diastolic BP of 10mmHg or more with symptoms.**   **A drop below systolic 90mmHg on standing.** | | | |
| **Continence** | | | | | | | | | |
| Urinary Incontinence **Y**  **N**  Nocturia **Y**  **___ N**  Urgency **Y**  **N**  Frequency **Y**  **N**  **Details:**  Faecal Incontinence **Y**  **N**  Constipation **Y**  **N**  Last bowel motion (specify):  Continence Wear: None  Wrap Around  Pull up  Slip in  Catheter **Y**  **N** | | | | | | | | | |
| **Functional Status** | **Previous Level of Function** | | | | | | | **Current Level of Function** | |
| **Functional Mobility Aid:** None  Walking Stick  Crutches  WZF  3-4 Wheel Voyager  Wheelchair | | | | | | | | | |
| Indoor |  | | | | | | |  | |
| Outdoor |  | | | | | | |  | |
| Stairs |  | | | | | | |  | |
| **Recent changes in mobility?** (specify) | | | | | | | | | |
| **Functional Status (contd)** | **Previous Level of Function (contd)** | | | | | **Current Level of Function (contd)** | | | |
| **Transfers** | | | | | | | | | |
| Chair | |  | | | | |  | | |
| Bed | |  | | | | |  | | |
| Bed Mobility | |  | | | | |  | | |
| Toilet / Commode | |  | | | | |  | | |
| Bath / shower | |  | | | | |  | | |
| **Personal ADL** | | | | | | | | | |
| Toileting / continence | |  | | | | |  | | |
| Wash (upper and lower body) | |  | | | | |  | | |
| Dress / groom (upper and lower body) | |  | | | | |  | | |
| Feeding | |  | | | | |  | | |
| **Domestic / Community ADL** | | | **Equipment In situ** | | | | | | |
| Meal Prep: | | | Wheelchair ________  Other Seating ________  Hoist  Transfer Aid ______  Hospital Bed  Pressure cushion _____  Bed Lever  Ortho Chair  RTS  Toilet Frame  Commode  Bath aid ________  Grab rail and position ____________  Other (specify) | | | | | | |
| Hot Drink Prep: | | |
| Shopping: | | |
| Finance: | | |
| Collecting Pension: | | |
| Other (housework, gardening, bins): | | |
| **Work or Leisure**: | | |
| **Comment**: | | | | | | | | | |
| **What Matters to You**? | | | | | | | | | |
| **Further Assessment/Collateral/Assessment Summary** | | | | | | | | | |
|  | | | | | | | | | |
| **Action Plan** | | | | | | | | | |
| **Problems Identified** | **Actions** | | | | | **Completed** | | | |
| **1**. |  | | | | |  | | | |
| **2**. |  | | | | |  | | | |
| **3**. |  | | | | |  | | | |
| **4**. |  | | | | |  | | | |
| **Intervention Record** | | | | | | | | | |
| **Medical Social Worker**: | | | | | | | | | |
| **Dietetics**: | | | | | | | | | |
| **Occupational Therapy**: | | | | | | | | | |
| **Physiotherapy**: | | | | | | | | | |
| **Speech and Language Therapy**: | | | | | | | | | |
| **Pharmacy**: | | | | | | | | | |
| **Advanced Nurse Practitioner**: | | | | | | | | | |
| **Registrar**: | | | | | | | | | |
| **Consultant**: | | | | | | | | | |
| **Patient Plan of Care** | | | | | | | | | |
| **Disciplines required in ED or Admission:** | **Discharge to:** | | | | | **Collateral History:** | | | |
| Medical Social Worker (MSW)  Physiotherapy (PT)  Occupational Therapy (OT)  Dietician  Speech and Language Therapy (SLT)  Pharmacy  ANP (specify specialty):  CNS (specify specialty):  Tissue Viability Nurse (TVN)  Geriatrician  Others (specify): | Admitted to Hospital Time:  Home  Nursing Home  Rehabilitation Site:  Respite  Public Health Nurse  Community Intervention Team (CIT)  General Practitioner (GP)  Integrated Care Team Older Persons(ICT)  Day Hospital  Smithfield Rapid Injury Clinic  Primary Care Team (specify)  CRT / Reablement | | | | | Family / Carer  GP  Others  Nursing Home | | | |
| **Assessor name and designation:** | | | | | **Date:** | | | | **Time:** |
