# Supplementary figures and images for: Application of the world guidelines for falls prevention and management’s risk stratification algorithm to patients on a frailty intervention pathway and the potential utility of sensory impairment information
Source: BMC Geriatr. 2024 Oct 12;24:824. doi: 10.1186/s12877-024-05405-3 (PMC11470725; doi:10.1186/s12877-024-05405-3)

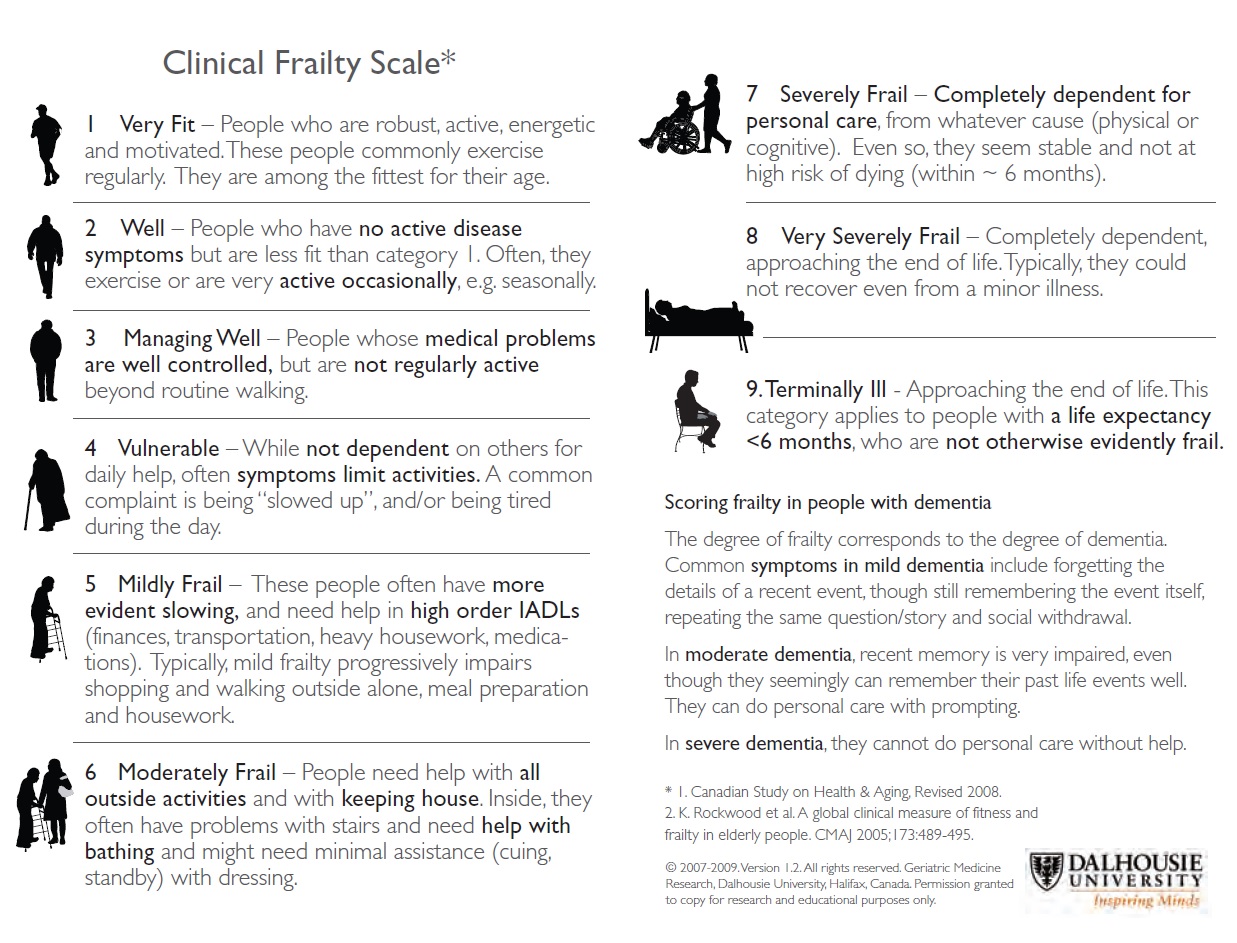

Supplement: Supplementary file 2 — Supplementary Material 2 [file 12877_2024_5405_MOESM2_ESM.docx]
