# Supplementary material for: Application of the world guidelines for falls prevention and management’s risk stratification algorithm to patients on a frailty intervention pathway and the potential utility of sensory impairment information
Source: BMC Geriatr. 2024 Oct 12;24:824. doi: 10.1186/s12877-024-05405-3 (PMC11470725; doi:10.1186/s12877-024-05405-3)
Supplement: Supplementary file 3 — Supplementary Material 3 [file 12877_2024_5405_MOESM3_ESM.docx]

STROBE Statement—checklist of items that should be included in reports of observational studies

|  | Item No. | Recommendation | Page  No. | Relevant text from manuscript |
| --- | --- | --- | --- | --- |
| **Title and abstract** | 1 | (*a*) Indicate the study’s design with a commonly used term in the title or the abstract | 2 | “in this retrospective cross-sectional study” |
|  |  | (*b*) Provide in the abstract an informative and balanced summary of what was done and what was found | 2-3 | All abstract |
| Introduction | | | |  |
| Background/rationale | 2 | Explain the scientific background and rationale for the investigation being reported | 3-4 | First four paragraphs of introduction section |
| Objectives | 3 | State specific objectives, including any prespecified hypotheses | 5 | Last paragraph of introduction section |
| Methods | | | |  |
| Study design | 4 | Present key elements of study design early in the paper | 6 | Second paragraph of methods section |
| Setting | 5 | Describe the setting, locations, and relevant dates, including periods of recruitment, exposure, follow-up, and data collection | 6 | Second paragraph of methods section |
| Participants | 6 | (*a*) *Cohort study*—Give the eligibility criteria, and the sources and methods of selection of participants. Describe methods of follow-up  *Case-control study*—Give the eligibility criteria, and the sources and methods of case ascertainment and control selection. Give the rationale for the choice of cases and controls  *Cross-sectional study*—Give the eligibility criteria, and the sources and methods of selection of participants | 6 | Inclusion and exclusion criteria, second paragraph of methods section |
|  |  | (*b*) *Cohort study*—For matched studies, give matching criteria and number of exposed and unexposed  *Case-control study*—For matched studies, give matching criteria and the number of controls per case | N/A | N/A |
| Variables | 7 | Clearly define all outcomes, exposures, predictors, potential confounders, and effect modifiers. Give diagnostic criteria, if applicable | 6-7 | Second and third paragraphs of methods section |
| Data sources/ measurement | 8* | For each variable of interest, give sources of data and details of methods of assessment (measurement). Describe comparability of assessment methods if there is more than one group | 6-8 | Description of outcome measures and raw data collected in method section |
| Bias | 9 | Describe any efforts to address potential sources of bias | 6 | “all patients aged 65 years or older, who attended the emergency department at the Mater Misericordiae University Hospital and who had a CGA carried out by the FIT between January 3^rd^ and April 20^th^ 2023 were included in the study” |
| Study size | 10 | Explain how the study size was arrived at | 6 | “Following an assessment of a random two-week time window of the number of patients who had a CGA carried out, it was established that three months’ worth of data would be adequate accounting for patterns of missing data.” |

Continued on next page

| Quantitative variables | 11 | Explain how quantitative variables were handled in the analyses. If applicable, describe which groupings were chosen and why | N/A | N/A |
| --- | --- | --- | --- | --- |
| Statistical methods | 12 | (*a*) Describe all statistical methods, including those used to control for confounding | 8 | “Binomial logistic regression was used to assess the relationship between sensory variables (vision, hearing, dizziness, and balance), falls and falls risk stratification. The regression controlled for the effect of age, sex, and frailty. The statistical software R [19] was used to analyse the data.” |
|  |  | (*b*) Describe any methods used to examine subgroups and interactions | 8 | “The regression controlled for the effect of age, sex, and frailty.” |
|  |  | (*c*) Explain how missing data were addressed | 8 | “Missing data was coded N/A and patients with missing data for any given key criteria were excluded from the corresponding analysis” |
|  |  | (*d*) *Cohort study*—If applicable, explain how loss to follow-up was addressed  *Case-control study*—If applicable, explain how matching of cases and controls was addressed  *Cross-sectional study*—If applicable, describe analytical methods taking account of sampling strategy | 8 | “Missing data was coded N/A and patients with missing data for any given key criteria were excluded from the corresponding analysis.” |
|  |  | (*e*) Describe any sensitivity analyses | N/A | N/A |
| Results | | | | |
| Participants | 13* | (a) Report numbers of individuals at each stage of study—eg numbers potentially eligible, examined for eligibility, confirmed eligible, included in the study, completing follow-up, and analysed | 8 | First paragraph of results section |
|  |  | (b) Give reasons for non-participation at each stage | N/A | N/A |
|  |  | (c) Consider use of a flow diagram | N/A | N/A |
| Descriptive data | 14* | (a) Give characteristics of study participants (eg demographic, clinical, social) and information on exposures and potential confounders | 8 | Table 1 |
|  |  | (b) Indicate number of participants with missing data for each variable of interest | 8 | First paragraph of results section |
|  |  | (c) *Cohort study*—Summarise follow-up time (eg, average and total amount) | N/A | N/A |
| Outcome data | 15* | *Cohort study*—Report numbers of outcome events or summary measures over time | N/A | N/A |
|  |  | *Case-control study—*Report numbers in each exposure category, or summary measures of exposure | N/A | N/A |
|  |  | *Cross-sectional study—*Report numbers of outcome events or summary measures | 8-9 | All results descriptions |
| Main results | 16 | (*a*) Give unadjusted estimates and, if applicable, confounder-adjusted estimates and their precision (eg, 95% confidence interval). Make clear which confounders were adjusted for and why they were included | 8-11 | Data analysis and results sections |
|  |  | (*b*) Report category boundaries when continuous variables were categorized | N/A | N/A |
|  |  | (*c*) If relevant, consider translating estimates of relative risk into absolute risk for a meaningful time period | N/A | N/A |

Continued on next page

| Other analyses | 17 | Report other analyses done—eg analyses of subgroups and interactions, and sensitivity analyses | 8 & 11 | Data analysis section and last section of results |
| --- | --- | --- | --- | --- |
| Discussion | | | | |
| Key results | 18 | Summarise key results with reference to study objectives | 11 | First paragraph of discussion section |
| Limitations | 19 | Discuss limitations of the study, taking into account sources of potential bias or imprecision. Discuss both direction and magnitude of any potential bias | 12-14 | Last three paragraphs of discussion section |
| Interpretation | 20 | Give a cautious overall interpretation of results considering objectives, limitations, multiplicity of analyses, results from similar studies, and other relevant evidence | 12-14 | Last three paragraphs of discussion section |
| Generalisability | 21 | Discuss the generalisability (external validity) of the study results | 14 | Conclusion section |
| Other information | |  | | |
| Funding | 22 | Give the source of funding and the role of the funders for the present study and, if applicable, for the original study on which the present article is based | 15 | Funding section |

*Give information separately for cases and controls in case-control studies and, if applicable, for exposed and unexposed groups in cohort and cross-sectional studies.

**Note:** An Explanation and Elaboration article discusses each checklist item and gives methodological background and published examples of transparent reporting. The STROBE checklist is best used in conjunction with this article (freely available on the Web sites of PLoS Medicine at http://www.plosmedicine.org/, Annals of Internal Medicine at http://www.annals.org/, and Epidemiology at http://www.epidem.com/). Information on the STROBE Initiative is available at www.strobe-statement.org.
